# Pan-cancer analysis of homologous recombination deficiency and homologous recombination repair–associated gene alterations in solid tumors from a large Asian cohort

**DOI:** 10.1186/s12885-025-14267-w

**Published:** 2025-05-26

**Authors:** Lili Ren, Runsi Yao, Ting Hou, Chenglin Liu, Fei Zhao, Xiaojun Chen, Zhou Zhang, Yan Huang

**Affiliations:** 1 Department of Medical Oncology, Affiliated Hospital of Hebei University, Hebei Key Laboratory of Cancer Radiotherapy and Chemotherapy, Baoding, China; 2 Department of Obstetrics, Shenzhen Second People’s Hospital, The First Affiliated Hospital of Shenzhen University, Shenzhen, China; 3 Burning Rock Biotech, Building 6, Phase 2, Standard Industrial Unit, No. 7 LuoXuan 4 th Road, International Biotech Island, Guangzhou, 510300 China; 4 Department of Oncology, Shanghai Medical College of Fudan University, 270 Dong-An Rd, Xuhui District, Shanghai, 200032 China; 5 Department of Gynecologic Oncology, Fudan University Shanghai Cancer Center, 270 Dong-An Rd, Xuhui District, Shanghai, 200032 China

**Keywords:** Biallelic alterations, Genomic instability, Homologous recombination deficiency, Homologous recombination repair, *TP53*

## Abstract

**Background:**

Homologous recombination deficiency (HRD) is associated with sensitivity to platinum-based chemotherapy and PARP inhibitors in BRCA-associated cancers**,** including ovarian, breast, prostate, and pancreatic cancers. This study explores HRD and homologous recombination repair (HRR) gene alterations in a pan-cancer cohort to guide precision oncology.

**Methods:**

Clinical and genomic data from 9,262 patients with 17 solid tumor types were analyzed using the OncoScreen^TM^ Plus kit. HRD scores, biallelic HRR and tumor suppressor gene alterations, and their clinical correlations were evaluated.

**Results:**

HRD scores varied across cancer types, all showing a long tail in distribution. The prevalence of pathogenic alterations in pan-cancer HRR was 21.3%, with 13.7% of the cases having an HRD score ≥42. HRD-related events (LOH, LST, and TAI) exhibited similarities and cancer-specific patterns at the chromosomal arm level. Biallelic loss of HRR genes, especially *BRCA1*, *BRCA2*, *RAD51D*, *RAD51 C*, and *PPP2R2 A* was linked to higher HRD scores in BRCA-associated cancers, while *BARD1*, *RAD51D*, *RAD54L*, *BRCA1*, and *MRE11* were associated with elevated HRD scores in in other cancer types (non-BRCA cancers). *TP53* biallelic alterations, with or without HRR alterations, were linked to increased HRD scores. Higher HRD scores were associated with late-stage, older, metastatic, PD-L1 positive, non-MSI-H/non-POLE samples were correlated with genomic instability indexes, such as structural chromosomal instability (SCIN), weighted genome instability index (WGII), and whole-genome doubling (WGD).

**Conclusions:**

This is the largest pan-cancer HRD study in an Asian population, providing insights for future HRD testing and targeted therapy.

**Supplementary Information:**

The online version contains supplementary material available at 10.1186/s12885-025-14267-w.

## Background

Cancer is a leading cause of mortality worldwide, with 2.4 million cancer-related deaths in China in 2020, where it has been the top cause of death since 2010 [1, 2]. Genomic instability, a hallmark of cancer, drives tumor progression, often triggered by DNA damage [3]. Homologous recombination repair (HRR) is the primary mechanism for repairing DNA double-strand breaks (DSBs), but homologous recombination deficiency (HRD), caused by loss-of-function mutations in HRR genes (e.g., *BRCA1* and *BRCA2*) or epigenetic inactivation, impedes accurate repair. This deficiency promotes reliance on error-prone pathways, increasing genomic instability and contributing to cancer pathogenesis [4]. Importantly, HRD sensitizes tumors to Poly ADP-ribose Polymerase (PARP) inhibitors, achieving synthetic lethality, and enhances responses to platinum-based therapies [5, 6].

Accurate HRD detection is crucial for precision oncology, though no unified standard exists. Current methods include: 1) Identifying HRD causes by detecting mutations or promoter methylation in HRR genes like *BRCA1*, *BRCA2* [7, 8]. 2) Assessing HRD effects through genomic damage patterns, including genome-wide loss of heterozygosity (gLOH), HRD score covering LOH, large-scale state transitions (LST), and telomeric allelic imbalance (TAI) [9, 10], alongside predictive models like SigMA [11] and HRDetect [12]. 3) Functional tests such as RAD51 foci formation assays, which evaluate HRR capability [13, 14].

Clinical trials have validated the effectiveness of PARP inhibitors, particularly in BRCA-associated cancers (ovarian, breast, prostate, and pancreatic cancers) [15–19]. In prostate cancer, patients with pathogenic alterations of 15 HRR genes show improved progression-free survival (PFS) [8], while in ovarian cancer, HRD-positive patients (HRD score ≥42 or gLOH ≥16%) benefit from PARP inhibitors [20–22]. Currently, genomic scar analysis based on Single Nucleotide Polymorphisms (SNPs) is the most promising clinical method for detecting HRD, effectively identifying potential beneficiaries of PARP inhibitor therapy among *BRCA* wild-type tumors [21].

HRD prevalence varies by tumor type, with BRCA-associated cancers showing the highest incidence, approximately 85% [23]. Beyond these cancers, HRD has clinical value in less common HRR-related cancers. For instance, small cell lung cancer patients carrying germline HRR mutations like *RAD51D*, *CHEK1*, *BRIP1*, *BRCA1/2* may experience LOH and potentially respond to PARP inhibitors [24]. Preclinical and early clinical studies indicate that PARP inhibitors may also be effective in gastrointestinal and genitourinary system cancers, extending beyond the current clinical application range [25, 26]. HRD’s potential as a biomarker for immunotherapy is being explored in ongoing trials [27, 28].

Pan-cancer analyses of HRD are gaining interest to explore associations with HRR genes and clinical characteristics across cancer types [29, 30]. Large-scale studies in diverse populations, especially Asian cohorts, remain limited. Most prior research has relied on The Cancer Genome Atlas (TCGA) or Western populations [29, 31]. A Korean study reported a 74.7% HRD incidence among 501 pan-cancer patients, higher than in Western cohorts [32]. Therefore, investigating HRR gene mutations and HRD status across diverse cancers in large Asian cohorts is essential. This study retrospectively analyzed 9,296 patients across 17 solid tumor types to explore HRD-related events, the influence of HRR and tumor suppressor gene status on HRD score, and the correlation between HRD scores and clinical genomic features.

## Materials and methods

### Patients and clinical data collection

This retrospective cross-sectional study collected clinical characteristics and genomic data from 9,262 patients with 17 solid tumor types tested using the OncoScreen^TM^ Plus panel (Burning Rock, Guangzhou, China) between January 2019 and December 2022. The panel covers 520 cancer-related genes and over 9000 SNP loci [33, 34]. Tumor tissues and matched whole blood samples were collected for parallel sequencing. All participants provided written informed consent for the use of their clinical information in research. The clinical characteristics of the patients used in analysis are summarized in Table 1. The median age of patients was 60 years, with 52.3% male and 44.1% female. Most patients were at advanced stages, with 20.5% in stage III and 53.4% in stage IV. Biopsies were primarily from primary lesions (72.8%), with smaller proportions from distant metastases (6.7%), lymph nodes (2.4%), and unknown sites (8.1%). Overall, 2% of patients were MSI-High (MSI-H). The most common cancers were lung, colorectal, stomach, ovarian, and breast cancer.

**Table 1:** Characteristics of 9262 patients from 17 solid tumor types

**Characteristic**	**Patients**	**LOH score** **median (interquartile range)**	**LST score** **median (interquartile range)**	**TAI score** **median (interquartile range)**	**HRD score** **median (interquartile range)**
Overall, n	9262	4 (1–7)	10 (4–17)	5 (2–9)	20 (8–33)
Age, mediamn (interquartile range)	60 (52–68)				
Sex					
Female, n (%)	4081 (44.1)	4 (1–7)	10 (4–18)	4 (2–8)	19 (7–33)
Male, n (%)	4842 (52.3)	5 (2–8)	10 (5–17)	5 (2–9)	21 (10–33)
Unknown, n (%)	339 (3.7)	3 (0–6.5)	8 (3–15)	4 (1–7)	15 (4–30)
MSI status					
MSI-H, n (%)	181 (2.0)	1 (0–1)	2 (1–3)	1 (0–2)	4 (1–7)
Not MSI-H, n (%)	9081 (98.0)	4 (1–8)	10 (4–17)	5 (2–9)	20 (9–33)
Stage					
I, n (%)	923 (10.0)	2 (0–5)	5 (1–11)	2 (0–5)	9 (2–20)
II, n (%)	744 (8.0)	3 (1–6)	8 (3–15)	4 (1–7)	16 (5–28)
III, n (%)	1900 (20.5)	5 (2–8)	11 (5–19)	5 (2–10)	21 (10–36)
IV, n (%)	4948 (53.4)	5 (2–8)	11 (5–18)	5 (2–9)	22 (11–34)
Unknown, n (%)	747 (8.1)	4 (1–7)	9 (3–16)	4 (1–8)	17 (7–31)
Sample source					
Primary, n (%)	6743 (72.8)	4 (1–7)	10 (4–17)	5 (2–8)	19 (7–32)
Metastatic, n (%)	619 (6.7)	5 (3–8)	12 (6–20)	6 (3–11)	25 (13–37)
Lymph node biopsy, n (%)	223 (2.4)	5 (3–8)	11 (6.5–18)	6 (3–10)	24 (14–37)
Unknown, n (%)	1677 (18.1)	4 (2–7)	10 (4–17)	5 (2–9)	19 (9–32)
Cancer Type					
Pan-cancer, n	9262	4 (1–7)	10 (4–17)	5 (2–9)	20 (8–33)
Lung, n	4852	4 (1–8)	10 (4–17)	5 (2–9)	21 (9–33)
Colorectum, n	1352	4 (2–6)	9 (4–13)	4 (2–6)	17 (10–25)
Stomach, n	622	3 (0–6)	9 (3–17)	4 (1–8)	17 (5–32)
Ovary, n	521	9 (3–14)	26 (9–34)	12 (5–15)	47 (18–63)
Breast, n	343	6 (3–10)	16 (7–26)	8 (3.5–12)	30 (13–47)
Pancreas, n	191	2 (0–6)	5 (0.5–12)	2 (0–6)	11 (1–24)
Biliary tract, n	190	5 (2–8)	10 (4.25–14)	4 (2–7.75)	20 (9–28.75)
Sarcoma, n	183	4 (1–9)	10 (3–21)	5 (2–10.5)	19 (6–41.5)
Endometrium, n	155	1 (0–2)	2 (0–5)	1 (0–3)	4 (0–9.5)
Kidney, n	142	2 (1–3)	3 (1–6)	2 (1–3)	7 (3–12)
Cervix, n	139	3 (1–5.5)	8 (4–13)	5 (2–7.5)	16 (9–26)
Liver, n	138	4 (2–7)	10 (6–14)	5 (3–8)	20.5 (13–28)
Prostate, n	108	4 (2–7)	11 (6–18.25)	3 (1–5)	19 (9.75–29)
Head and Neck, n	103	6 (2–8)	10 (4–16)	6 (2–10)	23 (10–35.5)
Esophagus, n	94	6 (3.25–9)	16 (10–23)	8 (5–11.75)	33 (19–41)
Bladder, n	65	4 (2–7)	13 (4–19)	4 (2–10)	22 (8–35)
Melanoma, n	64	5 (2.75–7)	10 (5–18.5)	6 (4–9)	20 (12.5–34.25)

### DNA large panel sequencing and data analyses

DNA extraction and sequencing were performed in Burning Rock Biotech, a CAP- accredited and CLIA-certified commercial clinical laboratory, according to optimized protocols as described previously [33]. Data analyses, including variants calling and interpretation, copy number variation, tumor mutational burden (TMB), and microsatellite instability (MSI) assessment, were carried out using standardized pipelines [34].

### Definition of pathogenic variation

HRR and tumor suppressor genes alterations were classified as pathogenic if they included frameshift, splice site, nonsense mutations, deep deletions and truncating rearrangements [29]. For missense, inframe insertion/deletion, and splice region variants, we referred to the ClinVar database [35] and used the CLNSIG or CLNSIGCONF indicators to define pathogenic variants. Specifically, variants with CLNSIG values of “Likely_pathogenic” or “Pathogenic” were considered pathogenic. Additionally, when CLNSIG was “Conflicting_interpretations_of_pathogenicity”, variants were also deemed pathogenic if CLNSIGCONF included “Likely_pathogenic” or “Pathogenic”.

### Define of biallelic and monoallelic alterations

Gene function is determined by the two allelic states at the same genetic locus on a pair of homologous chromosomes. LOH occurs when one of the allelic copies is lost, while deep loss occurs when both allelic copies are lost. Biallelic alterations were defined as two events: LOH with a germline or somatic mutation, a germline and a somatic mutation, two somatic mutations or deep loss. The LOH of gene was determined by analyzing the copy number of the chromosomal segment containing the gene, yielding two allelic copy numbers: the minor copy number and the major copy number. If the minor copy number was 0 and the major copy number was not 0, the gene was considered to have LOH event. If both the minor and major copy numbers were 0, the gene was considered to have a deep loss. The monoallelic alteration was defined by a pathogenic germline or somatic mutation.

### Genomic instability evaluation

HRD score was calculated as the sum of LOH, TAI, and LST, based on previously described methods [30]. Structural chromosomal instability (SCIN) was the sum of all structurally aberrant regions [36]. Regions of intrachromosomal gain and loss were defined relative to the modal copy number of the chromosome, and each region counted as one structural aberration. Weighted genome instability index (WGII) was the percentage of unstable chromosomal segments across all 22 chromosomes [36]. Whole-genome doubling (WGD) was defined when >50% of the autosomal genome exhibited an MCN (the more frequent allele in a given segment) ≥2 [37].

### The mutant-allele tumor heterogeneity (MATH)

MATH score was calculated by dividing the standard deviation of the mutant allele frequencies (MAFs) across all detected mutations by their mean and then multiplying by 100 [38].

### Immunohistochemical staining of PD-L1

PD-L1 staining was performed on FFPE samples using the 22 C3 antibody on the Dako automated platform, following 22 C3 PharmDx Assay protocol (Agilent, Santa Clara, CA, USA). Samples were considered positive if ≥5% of tumor cells exhibited moderate to strong membrane staining. Conversely, samples with <5% of cells stained were classified as negative [39, 40]. The tumor proportion score was used to express PD-L1 levels, with 0 indicating negative and ≥1% as positive. Two independent pathologists scored each slide.

### Statistical analysis

Data was analyzed using R version 4.2.2. The Chi-square test or Fisher’s exact test was used to compare categorical variables. Wilcoxon rank-sum test or Kruskal-Wallis test was used to compare continuous variables. Statistical significance was defined as a two-sided *P* value < 0.05.

## Results

### HRD score distribution and hotspot regions of HRD-related events

**Fig. 1:**
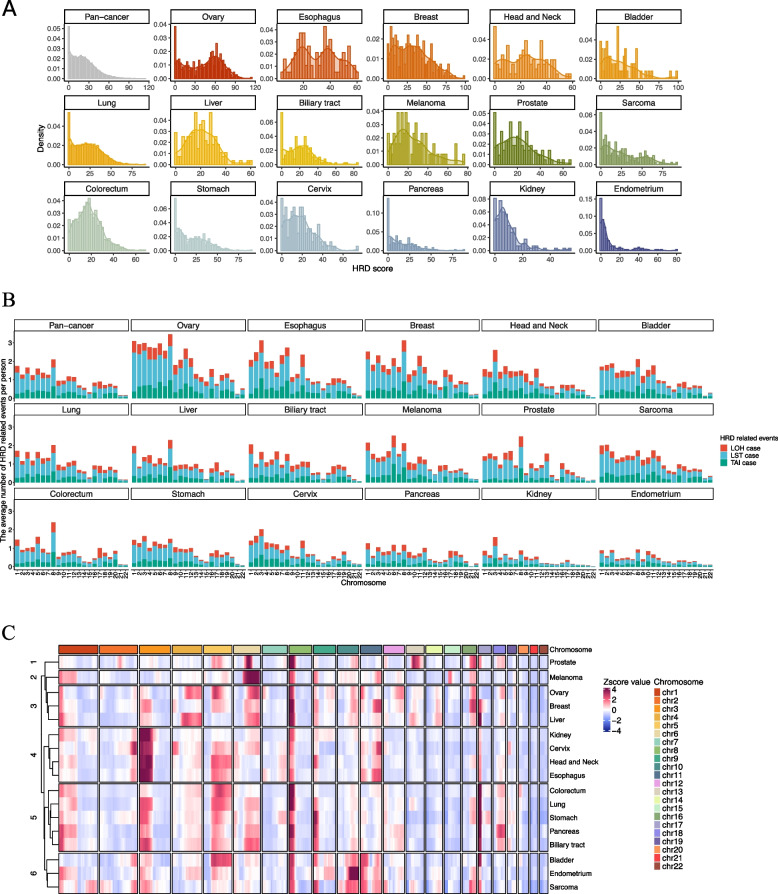
HRD score distribution and hotspot regions of HRD-related events. **A** The density distribution of HRD score across cancer types. **B** The average number of HRD-related(including LOH, LST, TAI) events across 22 autosomal chromosomes. **C** The heatmap of LOH events per 3-megabase interval on all 22 autosomal chromosomes

We examined HRD events across cancer types, ranking them by HRD scores (Table 1, Fig. 1A). Cancers with frequent BRCA mutations, like ovarian (median score: 47) and breast (30), exhibited higher HRD scores. Esophageal cancer ranked second with a median of 33, showing bimodal distribution. Kidney and endometrial cancers showed the lowest HRD scores. Compared to non-BRCA-associated cancers, pancreatic (11) and prostate (19) cancers did not exhibit significantly higher HRD scores. The long-tailed HRD score distribution suggests that some patients in each cancer type may benefit from PARP inhibitors or platinum-based chemotherapy.

LOH, LST, and TAI events were analyzed for their contribution to HRD and their chromosomal predilections (Fig. 1B). Overall, LST events were more prevalent than LOH and TAI across chromosomes. Chromosome (chr) 16 in breast, head and neck, and liver cancers showed high TAI rates; chr 18 in bladder and chr 6 in Melanoma also exhibited high TAI rates. Chr 17 in colorectal cancer showed frequent LOH. Chrs 1–8 had more HRD-related events, although this varied among cancer types. Compared to other chromosomes within the same cancer type, chr8 was more prone to HRD events in ovarian, breast, prostate, lung, liver, and colorectal cancers; chr 3 in kidney, head and neck, and esophagus cancers; and chrs 1, 6, and 8 in melanoma.

To assess similarities across cancer types, we calculated the number of HRD events per chromosome segment and transformed them into Z-scores for clustering (Fig. 1C, Figure S1). LOH events were frequently observed on chr 8p, consistent with the results in Fig. 1B. Kidney, cervix, head and neck, and esophageal cancers had high occurrence of LOH on chr 3p. Prostate and melanoma cancers had high LOH on chr 6q, while bladder, endometrium cancers, and sarcoma showed high LOH on chr 10q and chr 11p. Several cancers showed high LOH on chr 17p. Clustering results indicated that different cancers share similar chromosomal instability patterns, possibly reflecting their evolutionary processes. Additionally, LST, TAI, and overall HRD events also showed a degree of genomic instability similarity across cancer types (Supplementary figure 1).

### The distribution of HRR pathogenic alteration and biallelic status

**Fig. 2:**
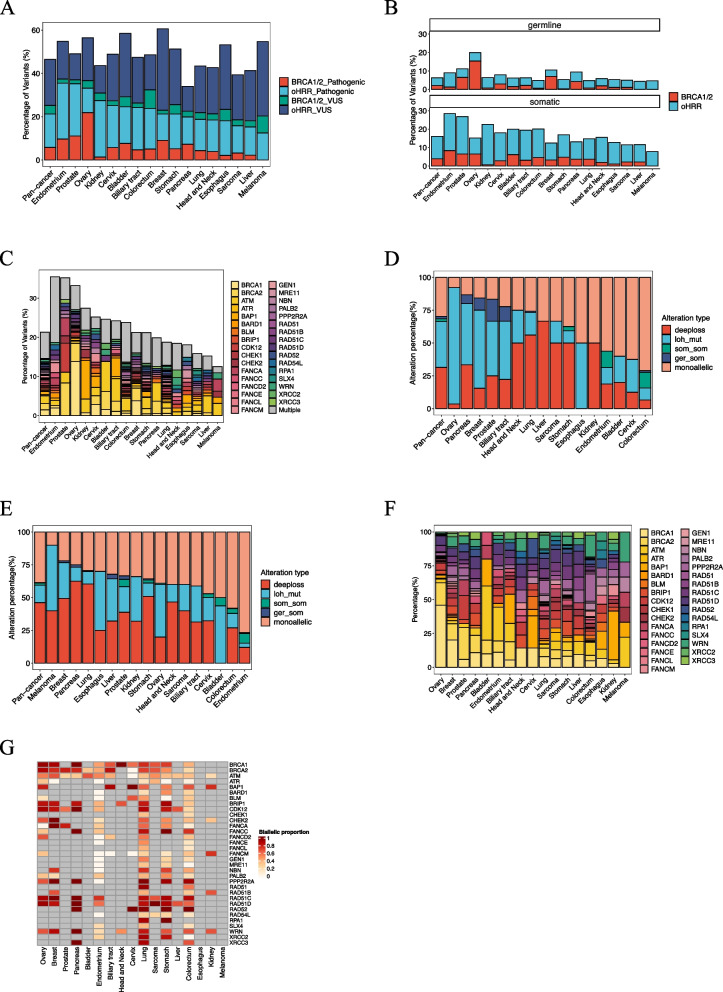
The distribution of HRR pathogenic alterations and biallelic status in various cancer types. **A** HRR gene alteration percentage. **B** Somatic and germline pathogenic alterations of HRR gene. **C** Pathogenic alterations of HRR gene in details. **D** *BRCA1/2* genes biallelic status. **E** oHRR genes biallelic status. **F** The percentage of HRR genes with biallelic alterations. **G** The proportion of biallelic alterations within each HRR gene alterations (HRR gene alterations in any cancer type with fewer than three occurrences were excluded)

HRR gene alterations, particularly *BRCA1/2*, are one of the primary causes of HRD. We classified HRR genes (as detailed in Supplementary table 1) into *BRCA1/2* and other HRR (oHRR) genes. HRR gene alterations were categorized into pathogenic and variants of unknown significance (VUS), prioritized as *BRCA1/2* > oHRR, pathogenic > VUS. Of 9,262 patients, 46.5% exhibited HRR alterations, with 5.8% having *BRCA1/2* pathogenic alterations and 15.5% having oHRR alterations (Fig. 2A). The highest proportions of HRR pathogenic alterations were in endometrial (35.5%), prostate (35.2%) and ovarian (33.2%) cancers. However, the proportions of *BRCA1/2* and oHRR alterations differed, with pathogenic *BRCA1/2* alterations at 9.7% vs 11.1% vs 21.9% and oHRR alterations at 25.8% vs 24.1% vs 11.3%, respectively. Melanoma showed no pathogenic BRCA1/2 mutations. Kidney cancer exhibited the highest proportion of oHRR pathogenic alterations at 26.1%.

In subsequent analyses, we exclusively focused on pathogenic alterations, and investigated germline and somatic HRR alterations (Fig. 2B). Overall, somatic HRR alterations were more common than germline (16.1% vs 6.2%). For *BRCA1/2*, somatic and germline alterations were 3.9% and 2.0%, while for oHRR, they were 12.1% and 4.2%. However, in ovarian cancer, germline alterations (20.0%) exceeded somatic ones (15.2%), with *BRCA1/2* germline alterations at 15.4%. In breast cancer, *BRCA1/2* germline alterations (7.0%) were more prevalent than somatic (3.2%). In liver cancer, all pathogenic *BRCA1/2* alterations were somatic.

Upon analyzing individual HRR gene alterations, significant variability was observed across cancer types (Fig. 2C). Multiple alterations were most frequent in endometrial (16.8%), colorectal (10.1%), and stomach (7.9%) cancers. *BRCA1* alterations were particularly common in ovarian cancer (13.8%), while *BRCA2* was prevalent in prostate cancer (8.3%). *BAP1* alterations stood out in kidney (9.9%) and cervix (2.9%) cancers, with *ATM* most frequent in bladder cancer (7.7%), *CDK12* notable in prostate cancer (7.4%), and *FANCA* prominent in melanoma (3.1%).

Biallelic inactivation of HRR genes, primarily caused by LOH with mutations or deep loss, was observed in 70.2% of *BRCA1/2* alterations (Fig. 2D). Not surprisingly, ovarian (92.2%), pancreatic (86.7%), breast (84.4%), and prostate (83.3%) cancers exhibited the highest rates of biallelic *BRCA1/2* alterations, indicating a strong link to biallelic gene loss. Interestingly, in ovarian cancer, *BRCA1/2* biallelic alterations were primarily due to gene LOH with germline or somatic alteration, while dual somatic or combinations alterations were rare. Liver and esophageal cancer exhibited a completely different biallelic inactivation pattern. Among patients with oHRR alterations, 61.4% had biallelic alterations, predominantly from deep loss (Fig. 2E). Remarkably, melanoma had the highest proportion of oHRR biallelic alterations (90%). Colorectal cancer exhibited the lowest and second-lowest rates of *BRCA1/2* and oHRR biallelic alterations, correlating with its lower HRD scores. Across cancer types, *BRCA1/2* biallelic alterations were major contributors to HRD, especially in ovarian (45.9%/16.5%), breast (20.2%/11.9%), pancreatic (10.9%/12.7%), and prostate (5.9%/23.5%) cancers (Fig. 2F). Additionally, *CDK12* (17.6%) and *FANCA* (11.8%) also played significant roles in prostate cancer. Genes like *BAP1* and *ATM* showed high biallelic loss in kidney and bladder cancers.

To further understand the cancer-specific tendencies of HRR biallelic loss, we excluded HRR gene alterations in any cancer type with fewer than three occurrences to avoid skewed results (Fig. 2G). *BAP1* showed high propensity for biallelic loss in cervical (100%), biliary tract (88.9%), ovarian (80.0%), kidney (76.5%) cancers. Meanwhile, *ATM* alterations exhibited biallelic alterations in multiple cancers, but the proportions were generally lower, except in breast and bladder cancers, both at 66.7%. Pancreatic cancer showed an exceptionally high biallelic loss in HRR genes, with *BRCA1*, *CDK12*, *FANCC*, *PPP2R2 A*, *RAD51 C*, *RAD51D*, *RAD52*, *WRN*, and *XRCC3* all presenting 100% biallelic loss. Cancer-specific patterns of biallelic loss were observed, highlighting the complexity and variability of HRR gene alterations across cancers.

### HRD correlation with biallelic alterations in HRR genes

**Fig. 3:**
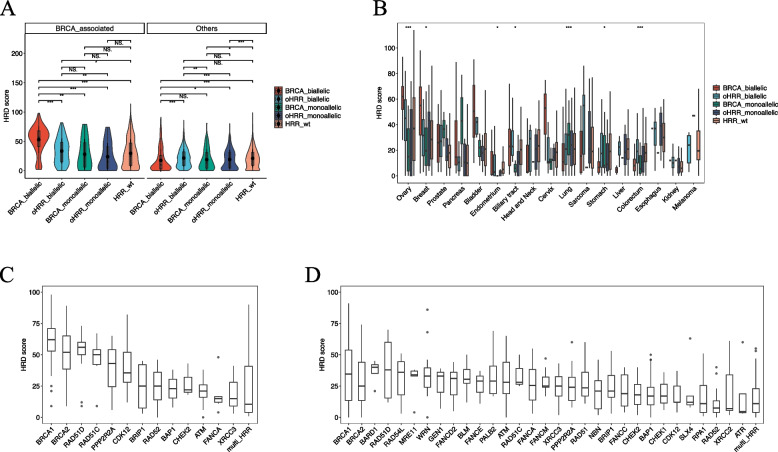
HRD score correlation with biallelic alterations in HRR genes. HRD score correlation with *BRCA1/2* and oHRR gene biallelic alteration in (**A**) BRCA-associated and other cancers and (**B**) each cancer type. HRD score with HRR gene biallelic alterations in (**C**) BRCA-associated and (**D**) other cancers. NS, not significant; *, *P*<0.05; **, *P*<0.01; ***, *P*<0.001

Traditionally, biallelic alterations in HRR genes are considered to have a decisive impact on HRD. We examined the effect of biallelic alterations in *BRCA1/2* and oHRR genes on HRD scores (Fig. 3A). As expected, in BRCA-associated cancers, patients with *BRCA1/2* biallelic alterations had significantly higher HRD scores than those with monoallelic alterations (*P*<0.01) and HRR wild-type patients (*P*<0.001). Furthermore, similar trends were observed in oHRR genes. Interestingly, patients with *BRCA1/2* biallelic alterations exhibited higher HRD scores than others with oHRR biallelic alterations (*P*<0.001), highlighting the greater impact of *BRCA1/2* on HRD in BRCA-associated cancers. However, in other cancers, oHRR biallelic alterations exhibited significantly higher HRD scores than *BRCA1/2* biallelic alterations (*P*<0.001) and oHRR monoallelic alterations (*P*<0.001), but comparable to wild-type groups (*P*>0.05). Surprisingly, in colorectal, stomach, endometrial, and lung cancers, HRR wild-type patients displayed comparable or slightly higher HRD scores than other alteration types (Fig. 3B).

We also investigated whether different HRR genes have a similar impact on the magnitude of HRD scores (Fig. 3C and D). In BRCA-associated cancers, biallelic alterations in *BRCA1* (median HRD score: 62), *BRCA2* (52), *RAD51D* (56), *RAD51C* (50), *PPP2R2A* (43), and *CDK12* (35.5) had higher HRD scores. In other cancers, *BARD1* (40), *RAD51D* (38), *RAD54L* (36), *BRCA1* (34.5), *MRE11* (34), *WRN *(33), *GEN1* (33), *FANCD2* (31), and *BLM* (30.5) showed higher HRD scores. The HRD scores for *BRCA1* or *BRCA2* were not significantly higher than other oHRR biallelic losses, consistent with the findings in Fig. 3A, indicating the possible influence of sample sizes and other factors in other cancers.

### HRD correlation with biallelic alterations in tumor suppressor genes

**Fig. 4:**
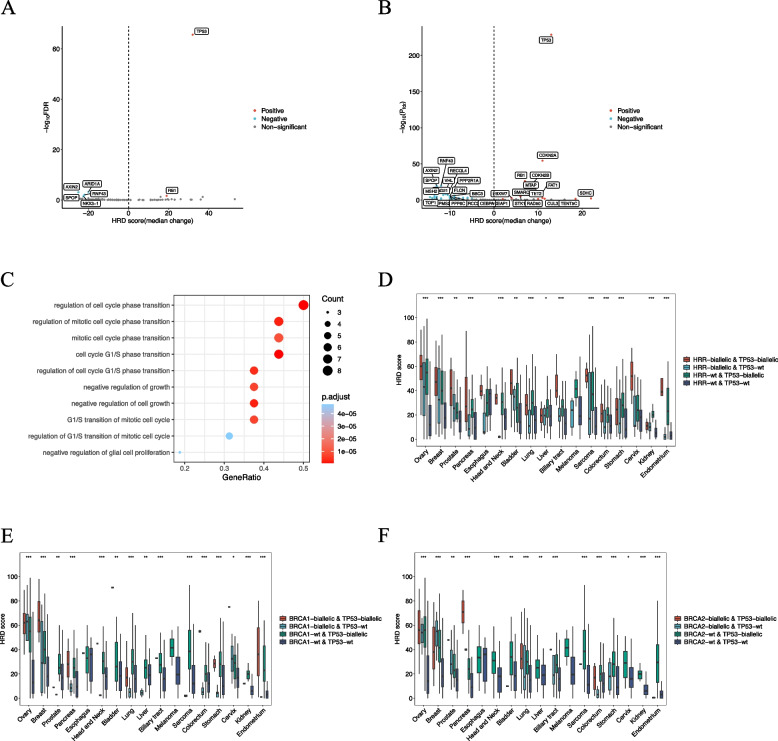
HRD score correlation with biallelic alterations in tumor suppressor genes. HRD score correlation with tumor suppressor gene biallelic alterations in (**A**) BRCA-associated and (**B**) other cancers. **C** GO enrichment analysis of genes capable of upregulating HRD scores. Effects of biallelic status of *TP53* with (**D**) HRR, **E** *BRCA1* and (F)*BRCA2* on HRD score. FDR, false discovery rate; NS, not significant; wt, wild type; *, *P*<0.05; **, *P*<0.01; ***, *P*<0.001

To explore factors influencing HRD beyond HRR gene alterations, we analyzed non-HRR tumor suppressor genes (Supplementary table 2 and Fig. 4). As anticipated, BRCA-associated cancers had fewer non-HRR tumor suppressor genes correlated with HRD scores compared to other cancers (7 vs 51, FDR<0.05). Across all cancer types, biallelic loss of *TP53* and *RB1* was associated with increased HRD scores (difference in HRD score between biallelic alteration and wild-type groups) (Fig. 4A and B). Conversely, *SPOP*, *AXIN2*, *RNF43*, and *ARID1 A* significantly correlated with decreased HRD scores. In other cancers, biallelic loss of genes such as *SDHC*, *TENT5 C*, *CUL3*, *CDKN2 A*, *CDKN2B*, *FAT1*, *SMARCA4*, *STK11*, and *KEAP1* was highly correlated with increased HRD scores, while *TOP1*, *DNMT3B*, *ASXL1*, *PTPRT*, *SDHA*, *RAD21*, and mismatch repair (MMR) core genes (*MSH2*, *PMS2*, *MSH6* and *MLH1*) correlated with decreased HRD scores. GO enrichment analysis of genes upregulating HRD scores revealed clustering in cell cycle and growth processes (Fig. 4C).

We further explored how co-alteration of HRR and tumor suppressor genes impacts HRD across cancers, focusing on combinations of *BRCA1*, *BRCA2*, other HRR genes, and *TP53* (Fig. 3D-F). A synergistic effect was observed between HRR and *TP53*, with patients having biallelic alterations in both genes exhibiting the highest HRD scores across most cancer types. Due to limited sample sizes for single gene alterations, similar phenomena were also observed for *BRCA1* and *BRCA2* in some cancers. Interestingly, in cancers like ovarian, breast, pancreatic, head and neck, lung, biliary tract, colorectal, stomach, endometrial, kidney, and sarcoma, patients with only *TP53* biallelic alterations had higher HRD scores than those with only HRR biallelic alterations, highlighting *TP53*’s strong influence on HRD.

### HRD correlation with clinical genomic features

**Fig. 5:**
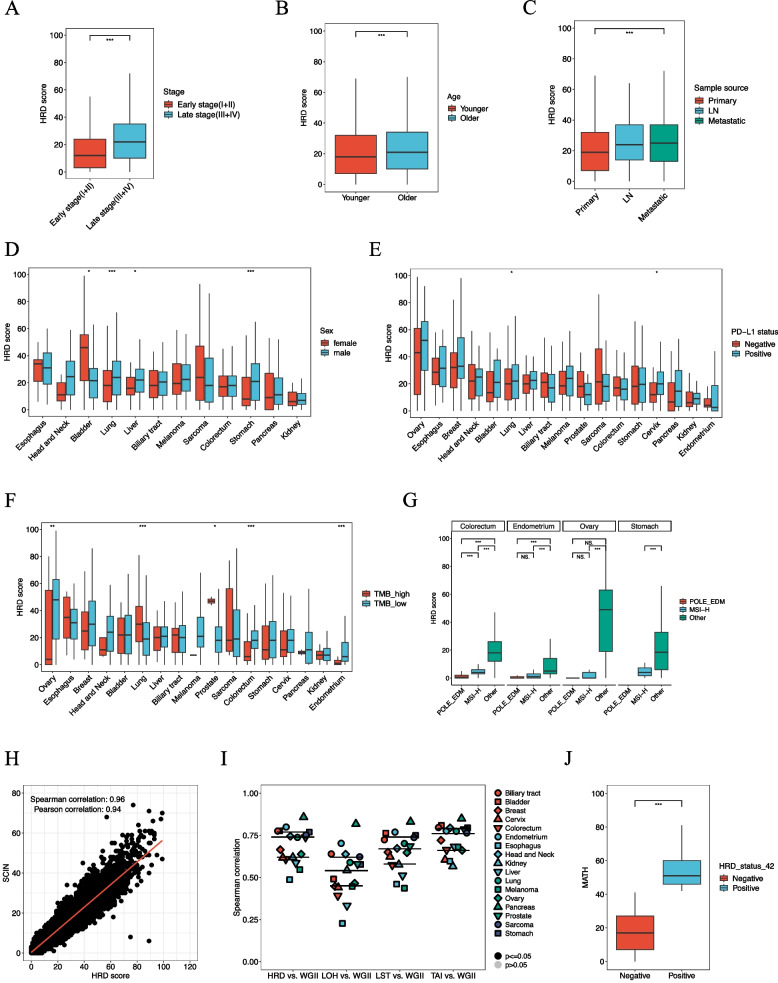
HRD score correlation with clinical genomic features. HRD correlation with (**A**) Stage, **B** Age, **C** Sample source, **D** Sex, **E** PD-L1, **F** TMB, **G** POLE and MSI-H, **H** SCIN, **I** WGII and (**J**) MATH. *, *P*<0.05; **, *P*<0.01; ***, *P*<0.001

HRD score reflects tumor characteristics, and we explored its correlation with clinical characteristics and molecular biological features (Fig. 5 and Supplementary figure 2). Late-stage (III and IV) tumors had higher HRD scores than early-stage (I and II) ones (median: 22 vs 12, *P*<0.001) (Fig. 5A and Supplementary figure 2A). Older patients generally had higher HRD scores (median: 21 vs 18, *P*<0.001), although in esophageal, breast, liver, and pancreatic cancers, younger patients showed higher HRD scores, though not statistically significant (Fig. 5B and Supplementary figure 2B). Metastatic and lymph node samples had higher HRD scores than primary tumors (25 vs 24 vs 19, *P*<0.001), likely reflecting stage differences (Fig. 5C and Supplementary figure 2C). Gender-specific differences included higher HRD scores in female bladder cancer and male lung, liver, and stomach cancers (Fig. 5D). Among samples with detailed documented histological subtypes, squamous cell carcinoma had higher HRD scores than adenocarcinoma samples in lung and cervical cancers (Supplementary figure 2D and E).

PD-L1 and TMB, biomarkers for immune checkpoint inhibitors, correlated with HRD scores. PD-L1 positivity was associated with higher HRD scores in most cancers, especially lung and cervical cancers (*P*<0.05) (Fig. 5E). Notably, this trend persisted even after excluding MSI-high or POLE-mutated tumors (Supplementary figure 2F). TMB-H correlated with higher HRD scores in lung and prostate cancers but lower scores in ovarian, colorectal, and endometrial cancers (Fig. 5F and Supplementary figure 2G). MSI-H and *POLE* EDM mutations were linked to lower HRD scores (*P*<0.001), showing a negative correlation in ovarian, colorectal, stomach, and endometrial cancers (Fig. 5G). After excluding MSI-H and *POLE* cases, TMB showed a positive correlation with HRD score (Supplementary figure 2H). TCGA data for COAD, READ, STAD, and UCEC were analyzed using the median TMB as a cutoff. Before excluding MSI-high and POLE-mutated tumors, TMB-low groups showed higher HRD scores in COAD (*P*=0.005) and UCEC (*P*<0.001) (Supplementary figure 2I). After exclusion, TMB-high groups had higher HRD scores, with significance in STAD (*P*<0.001) and UCEC (*P*<0.001) (Supplementary figure 2J). These results aligned with the study’s findings, confirming the TMB-HRD association’s consistency across platforms. After accounting for potential confounding by TP53 biallelic status, ovarian, colorectal, and endometrial cancers in the TP53 wild-type group showed significantly higher HRD scores in TMB-low samples (*P*<0.001, *P*<0.001, and *P*=0.001, respectively) (Supplementary figure 2K). However, the negative correlations observed in these cancers were no longer significant after excluding MSI-high and POLE-mutated tumors (Supplementary figure 2L).

HRD score, indicators of genomic instability, were highly correlated with SCIN (ρ = 0.96, *P*<0.001, Fig. 5H) and variably with WGII across cancers (median ρ = 0.74, interquartile range: 0.61–0.77, Fig. 5I). LOH had lower consistency with WGII compared to LST and TAI (0.53 vs 0.67 vs 0.76), with pancreatic cancer showing the highest correlation. WGD-positive patients consistently had higher HRD scores across cancers (Supplementary figure 2F).

Using an HRD score cutoff of 42, we classified patients as HRD-positive or negative, with a pan-cancer positivity rate of 13.7%. HRD-positive patients had significantly higher MATH scores (47.4 vs 33.5, *P*<0.001), indicating greater tumor heterogeneity and complexity (Fig. 5J and Supplementary figure 2G).

## Discussion

PARP inhibitors have been approved for HRD-positive ovarian cancer, offering benefits to *BRCA* wild-type but HRD-positive patients [21]. Concurrently, clinical trials targeting HRD-positive patients are underway in breast cancer (NCT05656131, NCT05288127, NCT05656131, NCT05085626, NCT02789332), and pancreatic cancer (NCT04666740, NCT05442749). The utility of HRD extends beyond BRCA-associated cancers. Positive results have also been observed with PARP inhibitor and immunotherapy treatments in gastrointestinal, lung, and genitourinary cancers [25–28]. Clinical trials related to HRD in lung (NCT03377556), colorectal (NCT05201612) cancer, and pan-cancer (NCT06065059, NCT04983745, NCT04890613, NCT04826341) are currently in progress. Recognizing that HRD extends beyond BRCA-related cancers, our study critically assessed HRD and HRR gene traits across 17 cancer types to enhance precision oncology strategies.

Previous pan-cancer HRD studies have predominantly focused on Western populations, with Foundation Medicine and TCGA studies reporting HRR gene pathogenic alterations in 18.9% and HRD positivity (HRD score ≥42) in 17.5% of cases [29, 31]. In contrast, a Korea study observed a much higher HRD prevalence of 74.7% [32]. Our study, representing the largest HRD analysis in an East Asian cohort, revealed HRR pathogenic alterations in 21.3% and HRD-positive in 13.7% of cases. These findings underscore the population-specific HRD traits and highlight the need for tailored approaches to HRD-related treatment strategies in different ethnic groups.

The distribution of HRD scores reveals subsets of patients with high HRD scores across cancer types, suggesting potential candidates for PARP inhibitors or platinum-based chemotherapy. Although HRD scores are anticipated to correlate closely with total CNV counts, mirroring the inherent chromosomal instability unique to each cancer type, our analysis also delves deeper to reveal specific chromosomal region-related HRD events that carry distinct clinical implications. For instance, in colorectal cancer, a high incidence of LOH on chrs 8p and 17p aligns with previous findings [41, 42]. Notably, chr18q11.2-q12.1 loss emerged as a biomarker for bevacizumab response, with multicenter studies showing improved survival in patients harboring this alteration. Similarly, in breast cancer, frequent deletions on chrs 8p, 11q, 16q, and 17p corroborate findings from TCGA and METABRIC cohorts, where 17p loss is associated with poorer survival [43]. Intriguingly, HER2-low tumors with chr17p loss exhibited heightened sensitivity to HER2-ADC drug T-Ama, suggesting that regional HRD events may refine therapeutic stratification [43]. In gastric cancer, common LOH regions include chrs 1p, 3p, 4, 5q, 9p, 17p, 18q, and 19p [44]. Oligodendroglioma patients with chrs 1p and 19q co-deletion exhibit markedly better survival [45]. Investigating HRD-related event distributions across different cancers’ chromosomal regions is profoundly insightful. Combining global HRD metrics with chromosomal region-related HRD events helps clarify each patient’s unique genomic instability profile, offering a stronger basis for treatment recommendations. We anticipate further studies elucidating the relationship between chromosomal region HRD-related events and clinical outcomes.

In cell line experiments, inhibiting HRR genes results in varying genomic damage, positioning HRR gene alterations as predictors of HRD-targeted therapy responsiveness [46]. Clinically, only prostate cancer currently assesses HRR gene alterations beyond *BRCA1/2* for guiding PARP inhibitor treatment [8]. Our study revealed widespread HRR gene alterations across cancer types, with somatic pathogenic alterations predominating except in ovarian cancer, where germline alterations (20.0%) exceeded somatic ones (15.2%), reflecting its familial risk and aligning with TCGA findings [47]. Interestingly, endometrial cancer showed the highest prevalence of pathogenic HRR gene alterations, followed by prostate, ovarian, and kidney cancers, though the former two had lower HRD scores, suggesting a complex relationship between HRR gene alterations and HRD. Further, cancer-specific patterns of HRR gene biallelic loss, underscore the diversity of genes contributing to HRD across cancers, warranting further exploration of its mechanisms and pathogenesis.

In BRCA-associated cancers, biallelic inactivation of *BRCA1/2* markedly affects HRD score, while in non-BRCA cancers, other mechanisms are involved. Although our targeted sequencing didn’t capture HRR gene methylation, we observed a significant correlation between biallelic inactivation of tumor suppressor genes and HRD score, particularly in cell cycle-related processes. *TP53* biallelic loss, either alone or combine with HRR gene biallelic loss, may elevate HRD scores in various cancers. The relationship between *TP53* alterations, HRD, and genomic instability is complex. Some studies suggest that *TP53* alterations can activate the HRR pathway under certain conditions [48]. HRD-induced chromosomal instability (CIN) can promote tumor growth, yet excessive CIN reduces tumor cell viability [49]. *TP53* inactivation can lead to cell cycle errors and increase ploidy, helping tumor cells survive growth pressures and elevate HRD scores. In lung and prostate cancers, *TP53* alterations, including combined biallelic inactivation with other tumor suppressor genes, correlate with higher HRD scores, independent of HRR gene status [30, 50]. These findings highlight that *TP53*-driven CIN, beyond HRR pathway alterations, suggesting the need to consider tumor suppressor gene impacts and therapies targeting TP53 pathways when using HRD scores as biomarkers for PARP inhibitor therapy [51, 52].

Given the roles of PD-L1, TMB, and MSI-H in immunotherapy response, we examined their association with HRD score. Patients with PD-L1 positive showed elevated HRD scores, indicating potential benefits from combining PARP inhibitors with immunotherapy. The relationship between TMB and HRD varied by cancer type, due to the negative correlation of MSI-H and *POLE* alterations with HRD scores, consistent with TCGA and Westphalen’s analyses. In TCGA, MSI and HRD score were negatively correlated in endometrial, stomach, and colorectal cancers [47], while gLOH and MSI were mutually exclusive in Westphalen’s cohort [29]. After excluding MSI-H and *POLE* cases, a positive correlation between TMB and HRD score emerged in our cohort and our TCGA validating dataset, aligning with TCGA findings where g*BRCA1/2* mutations with LOH (used as a proxy for HRD positivity) exhibited higher TMB than those without LOH, suggesting HRD-positive patients could benefit from immunotherapy [53, 54].

Genomic instability, a hallmark of cancer, manifests as chromosomal arm or cytoband gains/losses, HRD, aneuploidy, dysregulated gene expression, and disrupted signaling pathways, contributing to advanced tumor stages, metastasis, poor survival, and therapy resistance [55, 56]. Copy number variations have been utilized in cancer subtyping and early detection for endometrial, gastric, and colorectal cancers [57–60]. Our research highlights HRD-related event distributions across cancers and explores the association between HRD score and genomic instability markers such as WGD, SCIN, and WGII, revealing consistent patterns with some cancer-specific nuances. These findings support HRD-based stratification to expand precise medicine benefit.

While our study is the largest HRD pan-cancer research in an Asian cohort, several limitations should be acknowledged. The sample size for certain cancers was relatively small, potentially underrepresenting their HRR and HRD characteristics. This limitation also restricted our ability to explore the impact of biallelic alterations in other tumor suppressor genes beyond *TP53* on HRD scores. Additionally, the small sample size in some cancer types may have contributed to non-significant results, leaving open the question of whether this reflects a true absence of associations or merely a lack of statistical power. Moreover, due to the real-world clinical testing system from which our data were derived, inconsistent recording practices resulted in incomplete or non-uniform annotation of histological subtypes for many samples. This affected our ability to conduct detailed analyses of histological subtypes, which could have provided deeper insights into the relationship between HRD scores and specific tumor biology. Furthermore, our cohort includes samples from various disease stages, where prior treatments may have influenced HRD status. The HRD score reflects genomic history rather than current mutational processes, potentially leading to false positives if reversion alterations restoring HRR function or new HRD events occur undetected. Finally, the lack of clinical outcome data limits our ability to directly link HRD features to treatment outcomes, highlighting the need for further clinical studies to validate the HRD score’s applicability across different settings. Future studies with larger, more uniformly annotated cohorts are essential to address these limitations and to fully elucidate the role of HRD across different cancer types and settings.

## Conclusion

In summary, this study is the largest pan-cancer HRD analysis in an Asian population, offering a comprehensive view of HRD across multiple cancers. It paves the way for new opportunities in HRD detection and the development of targeted therapies.

## Supplementary Information

Supplementary Material 1: Supplementary Table 1. HRR genes list. Supplementary Table 2. Tumor suppressor genes list. Supplementary Figure 1. Heatmap of HRD, LST and TAI events on 22 chromosomes. Heatmap of (A) HRD, (B) LST, and (C) TAI events per 3-megabase interval on 22 autosomal chromosomes. Supplementary Figure 2. HRD correlation with clinical genomic features. HRD correlation with (A) Stage, (B) Age, (C) Sample source, (D) histological subtype in lung cancer, (E) histological subtype in cervical cancer, (F) PD-L1 status after excluding MSI-H and POLE-mutated tumors, (G) TMB, (H) TMB after excluding MSI-H and POLE-mutated tumors, (I) TMB in TCGA dataset, (J) TMB after excluding MSI-H and POLE-mutated tumors in TCGA dataset, (K) TP53 biallelic status and TMB, (L) TP53 biallelic status and TMB after excluding MSI-H and POLE-mutated tumors, (M) WGD, and (N) MATH.

## Data Availability

The datasets generated and/or analysed during the current study are not publicly available due to the large volume of data generated in this project and its ongoing use in further research, but are available from the corresponding author on reasonable request.

## Abbreviations

CEAC: Adenocarcinoma
Cervical CESC: Cervical squamous cell carcinoma
Chr: Chromosome
CIN: Chromosomal instability
DSB: DNA double-strand break
FFPE: Formalin-fixed paraffin-embedded
gLOH: Genome-wide loss of heterozygosity
HRD: Homologous recombination deficiency
HRR: Homologous recombination repair
LOH: Loss of heterozygosity
LST: Large-scale state transitions
LUAD: Lung adenocarcinoma
LUSC: Lung squamous cell carcinoma
MATH: Mutant-allele tumor heterogeneity
MMR: Mismatch repair
MSI: Microsatellite instability
MSI-H: Microsatellite instability-high
oHRR: Other HRR except for *BRCA1* and *BRCA2*
PARP: Poly ADP-ribose Polymerase
PFS: Progression-free survival
SCIN: Structural chromosomal instability
SCLC: Small cell lung cancer
SNP: Single nucleotide polymorphism
TAI: Telomeric allelic imbalance
TCGA: The Cancer Genome Atlas
TMB: Tumor mutational burden
VUS: Variants of unknown significance
WGD: Whole-genome doubling
WGII: Weighted genome instability index
