# Supplementary material for: Pan-cancer analysis of homologous recombination deficiency and homologous recombination repair–associated gene alterations in solid tumors from a large Asian cohort
Source: BMC Cancer. 2025 May 26;25:946. doi: 10.1186/s12885-025-14267-w (PMC12107993; doi:10.1186/s12885-025-14267-w)
Supplement: Supplementary file 1 — Supplementary Material 1: Supplementary Table 1. HRR genes list. Supplementary Table 2. Tumor suppressor genes list. Supplementary Figure 1. Heatmap of HRD, LST and TAI events on 22 chromosomes. Heatmap of (A) HRD, (B) LST, and (C) TAI events per 3-megabase interval on 22 autosomal chromosomes. Supplementary Figure 2. HRD correlation with clinical genomic features. HRD correlation with (A) Stage, (B) Age, (C) Sample source, (D) histological subtype in lung cancer, (E) histological subtype in cervical cancer, (F) PD-L1 status after excluding MSI-H and POLE-mutated tumors, (G) TMB, (H) TMB after excluding MSI-H and POLE-mutated tumors, (I) TMB in TCGA dataset, (J) TMB after excluding MSI-H and POLE-mutated tumors in TCGA dataset, (K) TP53 biallelic status and TMB, (L) TP53 biallelic status and TMB after excluding MSI-H and POLE-mutated tumors, (M) WGD, and (N) MATH. [file 12885_2025_14267_MOESM1_ESM.pdf]

Supplementary table 1. HRR genes list.

ATM	FANCA	RAD51
ATR	FANCC	RAD51B
BAP1	FANCD2	RAD51C
BARD1	FANCE	RAD51D
BLM	FANCL	RAD52
BRCA1	FANCM	RAD54L
BRCA2	GEN1	RPA1
BRIP1	MRE11	SLX4
CDK12	NBN	WRN
CHEK1	PALB2	XRCC2
CHEK2	PPP2R2A	XRCC3

Supplementary table 2. Tumor suppressor genes list.

APC	DIS3	KEAP1	PIK3R1	SMAD2	ARID5B
TP53	DNMT3A	KMT2A	PIK3R2	SMAD4	TENT5C
ARID1B	DNMT3B	KMT2C	PMS2	SMARCA4	CUL3
ARID2	EP300	KMT2D	POLD1	SMARCB1	BCL2L11
ASXL1	EPCAM	LATS1	POLE	SOCS1	CASP8
ASXL2	EPHA3	LATS2	PPP2R1A	SOX17	CBL
AXIN1	ERCC2	MAP2K4	PPP6C	AXIN2	CDC73
RB1	ERCC3	MAP3K1	PRDM1	STK11	CDKN2C
B2M	ERCC4	MAX	PRKN	SUFU	EED
BBC3	ERCC5	MEN1	PTCH1	TBX3	EPHB1
BCL10	ERRFI1	MGA	PTEN	TCF3	ID3
BMPR1A	ETV6	MLH1	PTPRD	TCF7L2	INHA
BTG1	FAS	MSH2	PTPRS	TET2	INPP4B
CBFB	FAT1	MSH3	PTPRT	TGFBR1	MST1
CDH1	FBXW7	MSH6	RAD21	TGFBR2	MYOD1
CDKN1A	FH	MTAP	RAD50	TMEM127	PARP1
CDKN1B	FLCN	MUTYH	RASA1	TNFAIP3	PHOX2B
CDKN2A	FOXO1	NCOR1	RNF43	TNFRSF14	PIK3R3
CDKN2B	FUBP1	NF1	RECQL4	TOP1	PMS1
CEBPA	GPS2	NF2	NKX3-1	SPOP	SDHB
CIC	GRIN2A	NFKBIA	RUNX1	TSC1	SDHD
CREBBP	HLA-A	ARID1A	SDHA	TSC2	SMAD3
CTCF	HLA-B	NPM1	SDHAF2	VHL	SPEN
CYLD	HLA-C	NTHL1	SETD2	ZNRF3	TET1
DAXX	HNF1A	PAX5	SH2B3	ABRAXAS1	
DICER1	IFNGR1	PBRM1	SHQ1	SDHC	

Supplementary figure 1. Heatmap of HRD, LST and TAI events on 22 chromosomes. Heatmap of (A) HRD, (B) LST, and (C) TAI events per 3-megabase interval on 22 autosomal chromosomes.

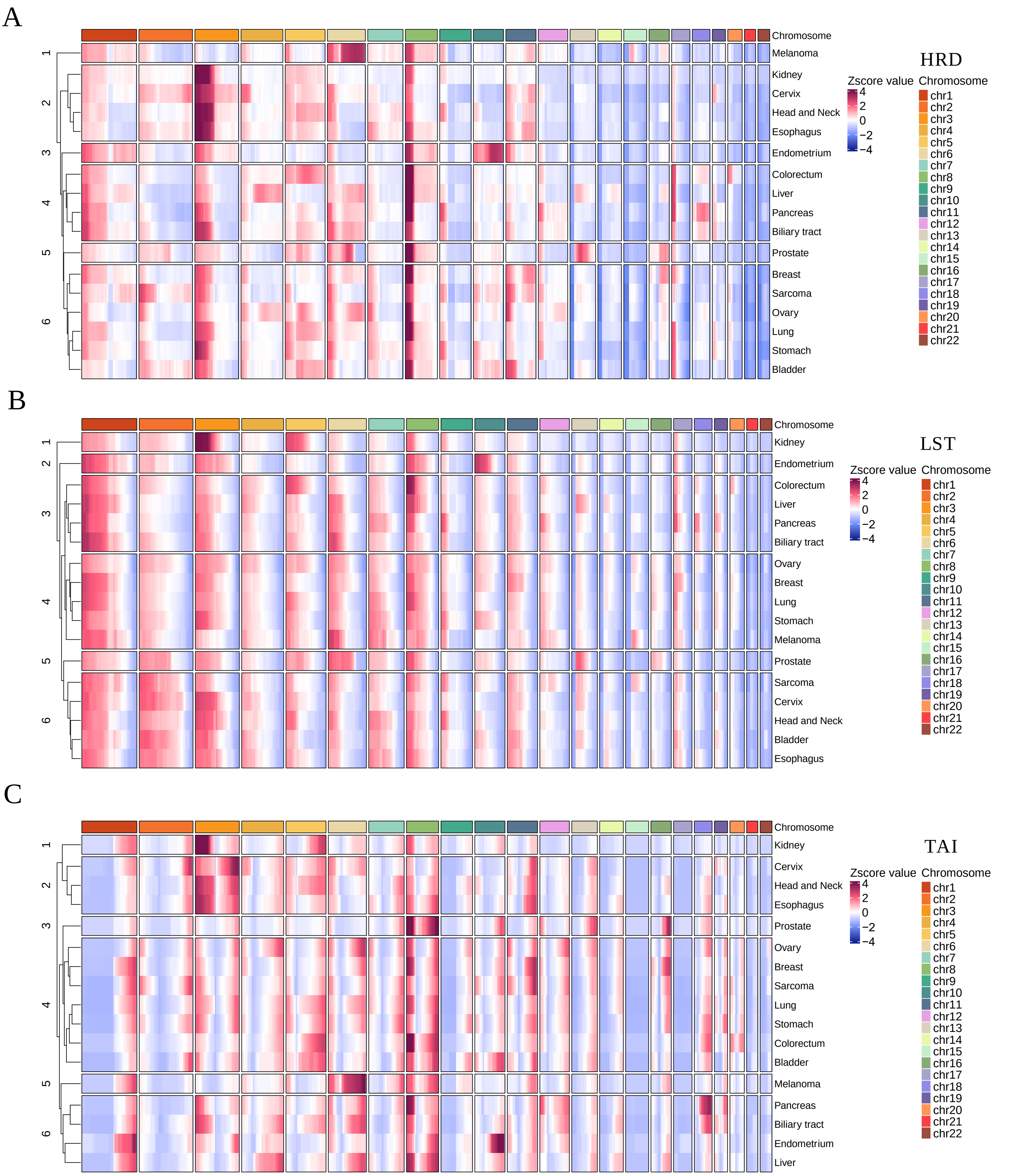

Supplementary figure 2. HRD correlation with clinical genomic features. HRD correlation with (A) Stage, (B) Age, (C) Sample source, (D) histological subtype in lung cancer, (E) histological subtype in cervical cancer, (F) PD-L1 status after excluding MSI-H and POLE-mutated tumors, (G) TMB, (H) TMB after excluding MSI-H and POLE-mutated tumors, (I) TMB in TCGA dataset, (J) TMB after excluding MSI-H and POLE-mutated tumors in TCGA dataset, (K) TP53 biallelic status and TMB, (L) TP53 biallelic status and TMB after excluding MSI-H and POLE-mutated tumors, (M) WGD, and (N) MATH.

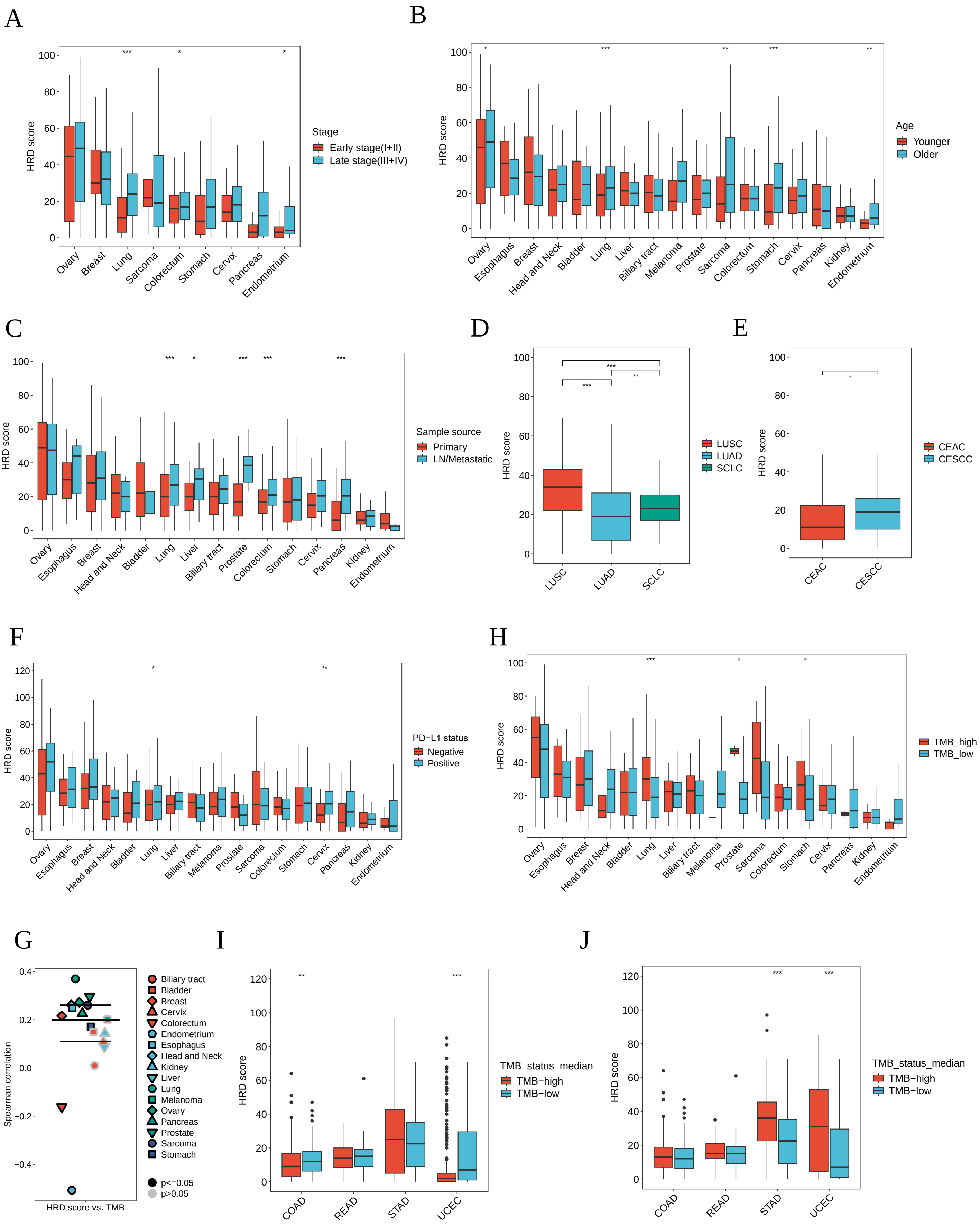

K

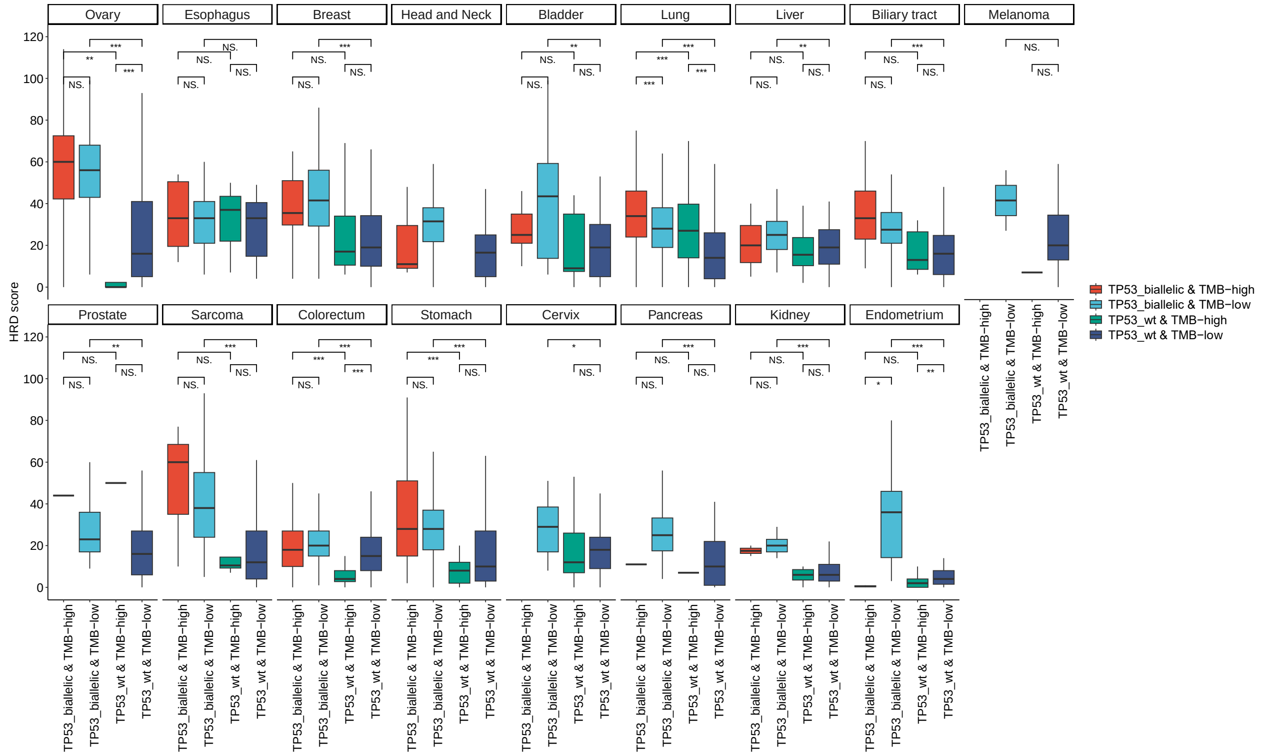
Ovary
Esophagus
Breast
Head and Neck
Bladder
Lung
Liver
Biliary tract
Melanoma
Prostate
Sarcoma
Colorectum
Stomach
Cervix
Pancreas
Kidney
Endometrium
HRD score
0
20
40
60
80
100
120
TP53_biallelic & TMB−high
TP53_biallelic & TMB−low
TP53_wt & TMB−high
TP53_wt & TMB−low
NS.
*
**
***

L

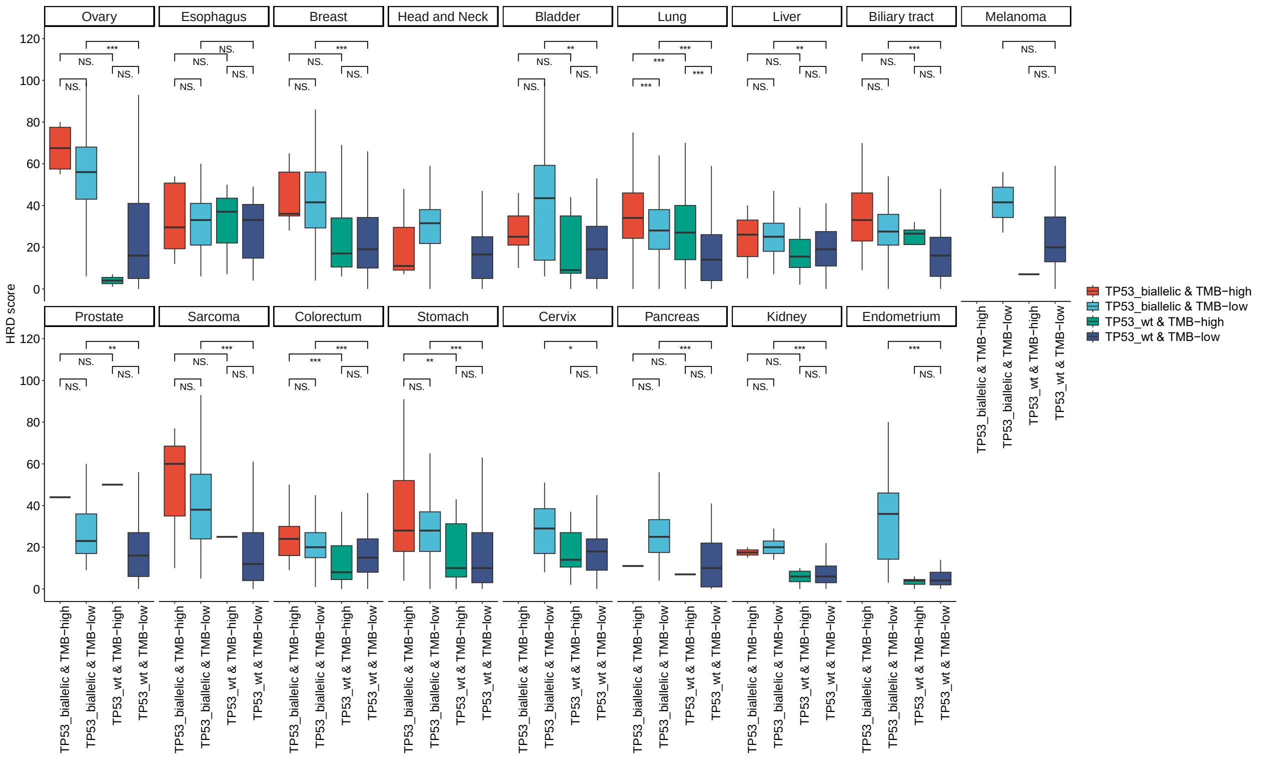
Ovary
Esophagus
Breast
Head and Neck
Bladder
Lung
Liver
Biliary tract
Melanoma
Prostate
Sarcoma
Colorectum
Stomach
Cervix
Pancreas
Kidney
Endometrium
HRD score
0
20
40
60
80
100
120
TP53_biallelic & TMB−high
TP53_biallelic & TMB−low
TP53_wt & TMB−high
TP53_wt & TMB−low
NS.
*
**
***

M

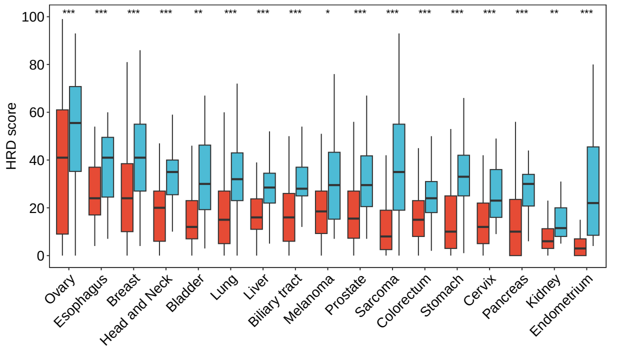
HRD score
0
20
40
60
80
100
WGD
Negative
Positive
Ovary
Esophagus
Breast
Head and Neck
Bladder
Lung
Liver
Biliary tract
Melanoma
Prostate
Sarcoma
Colorectum
Stomach
Cervix
Pancreas
Kidney
Endometrium

N

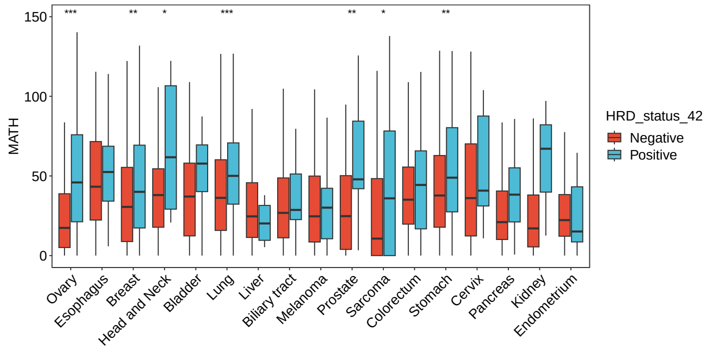
MATH
0
50
100
150
HRD_status_42
Negative
Positive
Ovary
Esophagus
Breast
Head and Neck
Bladder
Lung
Liver
Biliary tract
Melanoma
Prostate
Sarcoma
Colorectum
Stomach
Cervix
Pancreas
Kidney
Endometrium
